# (11*R*,13*R*)-13-(Tetra­lin-1-ylamino)-4,5-ep­oxy-11,13-dihydro­costunolide

**DOI:** 10.1107/S1600536808003322

**Published:** 2008-02-29

**Authors:** Shama Nasim, Sean Parkin, Peter A. Crooks

**Affiliations:** a Department of Pharmaceutical Sciences, College of Pharmacy, University of Kentucky, Lexington, KY 40536, USA; b Department of Chemistry, University of Kentucky, Lexington, KY 40506, USA

## Abstract

The title compound [systematic name: (12*R*)-4,8-dimethyl-12-[(1′*R*)-1′,2′,3′,4′-tetrahydro-1′-naphthyl)aminomethyl]-3,14-dioxatricyclo[9.3.0.0^2,4^]tetradec-7-en-13-one}, C_25_H_33_NO_3_, was formed from the reaction of (1*R*)-1-amino­tetra­lin with parthenolide in methano­lic solution. X-ray crystal structure analysis determined that the configuration of the new chiral center in the title compound was *R*.

## Related literature

For related literature, see: Allen *et al.* (1987 ▶); Crooks *et al.* (2005 ▶); Desiraju & Steiner (1999 ▶); Nasim *et al.* (2007*a* ▶,*b* ▶). 
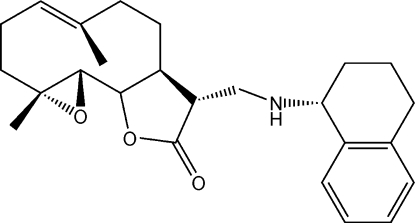


## Experimental

### 

#### Crystal data

C_25_H_33_NO_3_
*M* *_r_* = 395.52
Orthorhombic, 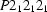

*a* = 8.4952 (13) Å
*b* = 13.1852 (19) Å
*c* = 18.771 (3) Å
*V* = 2102.6 (6) Å^3^
*Z* = 4
Cu *K*α radiation
μ = 0.64 mm^−1^
*T* = 90.0 (2) K
0.30 × 0.28 × 0.18 mm

#### Data collection

Bruker X8 Proteum diffractometer
Absorption correction: multi-scan (*SADABS* in *APEX2*; Bruker Nonius, 2004 ▶) *T*_min_ = 0.782, *T*_max_ = 0.894
26461 measured reflections
3849 independent reflections
3813 reflections with *I* > 2σ(*I*)
*R*_int_ = 0.033

#### Refinement

*R*[*F* ^2^ > 2σ(*F* ^2^)] = 0.043
*wR*(*F* ^2^) = 0.110
*S* = 1.06
3849 reflections
269 parameters
H atoms treated by a mixture of independent and constrained refinement
Δρ_max_ = 0.34 e Å^−3^
Δρ_min_ = −0.22 e Å^−3^
Absolute structure: Flack (1983 ▶), 1623 Friedel pairs
Flack parameter: 0.06 (5)

### 

Data collection: *APEX2* (Bruker Nonius, 2004 ▶); cell refinement: *APEX2*; data reduction: *APEX2*; program(s) used to solve structure: *SHELXS97* (Sheldrick, 2008 ▶); program(s) used to refine structure: *SHELXL97* (Sheldrick, 2008 ▶); molecular graphics: *XP* in *SHELXTL* (Sheldrick, 2008 ▶); software used to prepare material for publication: *SHELXL97* and local procedures.

## Supplementary Material

Crystal structure: contains datablocks global, I. DOI: 10.1107/S1600536808003322/hg2376sup1.cif

Structure factors: contains datablocks I. DOI: 10.1107/S1600536808003322/hg2376Isup2.hkl

Additional supplementary materials:  crystallographic information; 3D view; checkCIF report

## Figures and Tables

**Table 1:** Hydrogen-bond geometry (Å, °)

*D*—H⋯*A*	*D*—H	H⋯*A*	*D*⋯*A*	*D*—H⋯*A*
N1—H1*N*⋯O3	1.01 (3)	2.32 (3)	2.992 (2)	123.5 (18)

**Fig. 1.**
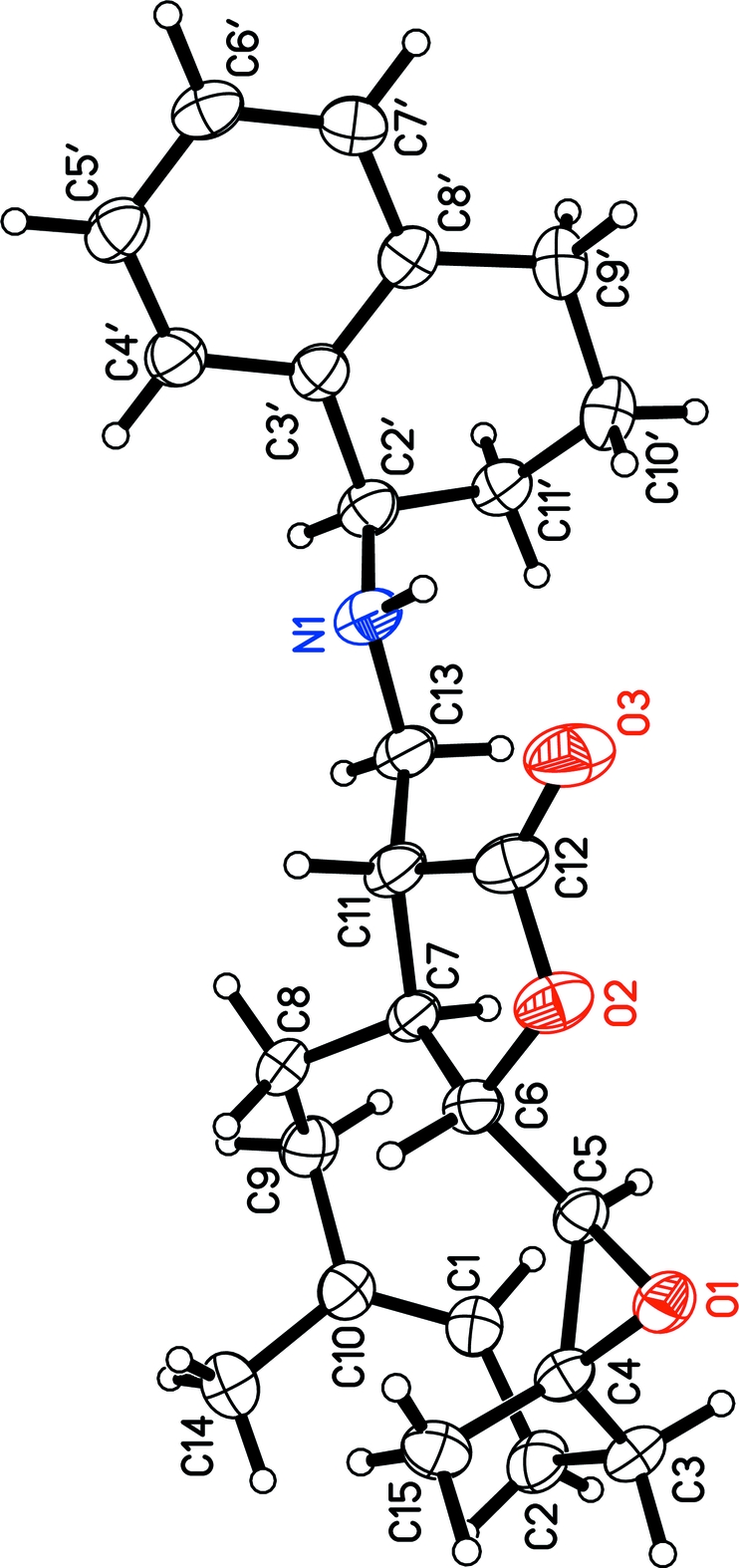
A view of the asymmetric unit of the title compound with displacement ellipsoids drawn at the 50% probability level; H atoms are shown as small spheres of arbitrary radii.

**Fig. 2.**
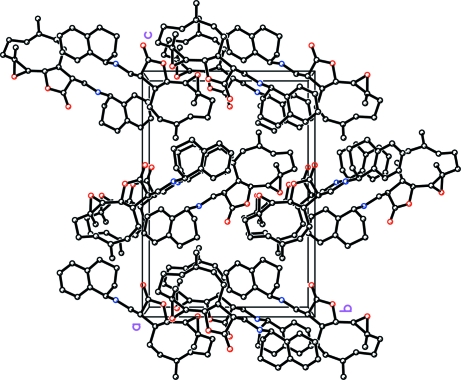
A packing diagram, viewed down the a axis, hydrogen atoms have been omitted for clarity.

## supplementary crystallographic information

### Comment

Due to the interesting biological activity of parthenolide, we have synthesized a series of amino analogs of parthenolide (Crooks *et al.*, 2005). In order to confirm the configuration of the newly formed methine carbon at C-11 in these molecules, and to obtain more detailed information on the structural conformation of the molecule that may be of value in structure activity relationship studies, the X-ray structure determination of the title compound has been carried out and the results are presented below. The absolute stereochemistry of the newly formed methine at C-11 was found to be *R*, which is typical in structurally related C-11 aminoparthenolide analogs that result from the reaction of an secondary amino compound with parthenolide (Nasim *et al.*, 2007*a*, 2007*b*). Bond distances and angles within the molecule were quite regular with normal bond lengths (Allen *et al.*, 1987). A hydrogen bond is observed between N-1H and O3 of the carbonyl oxygen of the 5-membered lactone ring (Desiraju *et al.*,1999) (2.32 (3) A°, 2.99 (2) A°, 123.5 (18)°) (Table 1).

### Experimental

The title compound was prepared utilizing the general procedure reported earlier (Nasim *et al.*, 2007*a*, 2007*b*).

### Refinement

H atoms were found in difference Fourier maps and subsequently placed in idealized positions with constrained C—H distances of 0.95Å (C_sp2_—H), 1.00Å (*R*_3_CH), 0.99Å (*R*_2_CH_2_), 0.98Å (RCH_3_) except for the NH hydrogen coordinates, which were refined. *U*_iso_(H) values were set to 1.2*U*_eq_ or 1.5*U*_eq_ (RCH_3_ only) of the attached atom.

### Crystal data

**Table:**

C_25_H_33_NO_3_	*F*(000) = 856
*M**_r_* = 395.52	*D*_x_ = 1.249 Mg m^−^^3^
Orthorhombic, *P*2_1_2_1_2_1_	Cu *K*α radiation, λ = 1.54178 Å
Hall symbol: P 2ac 2ab	Cell parameters from 9023 reflections
*a* = 8.4952 (13) Å	θ = 4.1–68.6°
*b* = 13.1852 (19) Å	µ = 0.64 mm^−^^1^
*c* = 18.771 (3) Å	*T* = 90 K
*V* = 2102.6 (6) Å^3^	Cut block, colourless
*Z* = 4	0.30 × 0.28 × 0.18 mm

### Data collection

**Table:**

Bruker X8 Proteum diffractometer	3849 independent reflections
Radiation source: fine-focus rotating anode	3813 reflections with *I* > 2σ(*I*)
Helios multilayer optics	*R*_int_ = 0.033
Detector resolution: 18 pixels mm^-1^	θ_max_ = 68.6°, θ_min_ = 4.1°
ω and φ scans	*h* = −10→10
Absorption correction: multi-scan (*SADABS* in *APEX2*; Bruker Nonius, 2004)	*k* = −15→15
*T*_min_ = 0.782, *T*_max_ = 0.894	*l* = −22→22
26461 measured reflections	

### Refinement

**Table:**

Refinement on *F*^2^	Hydrogen site location: inferred from neighbouring sites
Least-squares matrix: full	H atoms treated by a mixture of independent and constrained refinement
*R*[*F*^2^ > 2σ(*F*^2^)] = 0.043	*w* = 1/[σ^2^(*F*_o_^2^) + (0.0627*P*)^2^ + 0.7419*P*] where *P* = (*F*_o_^2^ + 2*F*_c_^2^)/3
*wR*(*F*^2^) = 0.110	(Δ/σ)_max_ < 0.001
*S* = 1.06	Δρ_max_ = 0.34 e Å^−^^3^
3849 reflections	Δρ_min_ = −0.22 e Å^−^^3^
269 parameters	Extinction correction: *SHELXL97* (Sheldrick, 2008), Fc^*^=kFc[1+0.001xFc^2^λ^3^/sin(2θ)]^-1/4^
0 restraints	Extinction coefficient: 0.0147 (7)
Primary atom site location: structure-invariant direct methods	Absolute structure: Flack (1983)
Secondary atom site location: difference Fourier map	Flack parameter: 0.06 (5)

### Special details

**Table:**

Geometry. All e.s.d.'s (except the e.s.d. in the dihedral angle between two l.s. planes) are estimated using the full covariance matrix. The cell e.s.d.'s are taken into account individually in the estimation of e.s.d.'s in distances, angles and torsion angles; correlations between e.s.d.'s in cell parameters are only used when they are defined by crystal symmetry. An approximate (isotropic) treatment of cell e.s.d.'s is used for estimating e.s.d.'s involving l.s. planes.
Refinement. Refinement of *F*^2^ against ALL reflections. The weighted *R*-factor *wR* and goodness of fit *S* are based on *F*^2^, conventional *R*-factors *R* are based on *F*, with *F* set to zero for negative *F*^2^. The threshold expression of *F*^2^ > σ(*F*^2^) is used only for calculating *R*-factors(gt) *etc*. and is not relevant to the choice of reflections for refinement. *R*-factors based on *F*^2^ are statistically about twice as large as those based on *F*, and *R*- factors based on ALL data will be even larger.

### Fractional atomic coordinates and isotropic or equivalent isotropic displacement parameters (Å^2^)

**Table:**

	*x*	*y*	*z*	*U*_iso_*/*U*_eq_	
N1	0.5361 (2)	0.68044 (12)	0.44880 (10)	0.0365 (4)	
H1N	0.597 (3)	0.6648 (19)	0.4042 (13)	0.044*	
O1	0.79418 (15)	0.18237 (9)	0.50676 (7)	0.0315 (3)	
O2	0.76245 (16)	0.38600 (10)	0.45744 (7)	0.0363 (3)	
O3	0.7062 (2)	0.50746 (12)	0.38018 (8)	0.0508 (4)	
C1	0.5083 (2)	0.23023 (14)	0.67140 (10)	0.0317 (4)	
H1	0.4217	0.2389	0.6400	0.038*	
C2	0.5720 (3)	0.12589 (15)	0.67700 (11)	0.0389 (5)	
H2A	0.4851	0.0776	0.6862	0.047*	
H2B	0.6468	0.1220	0.7173	0.047*	
C3	0.6562 (3)	0.09719 (14)	0.60747 (11)	0.0355 (4)	
H3A	0.7194	0.0351	0.6152	0.043*	
H3B	0.5770	0.0824	0.5702	0.043*	
C4	0.7615 (2)	0.18070 (13)	0.58256 (9)	0.0286 (4)	
C5	0.6906 (2)	0.25699 (13)	0.53651 (9)	0.0259 (4)	
H5	0.5768	0.2449	0.5260	0.031*	
C6	0.7381 (2)	0.36493 (13)	0.53249 (10)	0.0275 (4)	
H6	0.8375	0.3764	0.5598	0.033*	
C7	0.6115 (2)	0.43955 (12)	0.55609 (10)	0.0264 (4)	
H7	0.5066	0.4092	0.5449	0.032*	
C8	0.6106 (2)	0.47212 (14)	0.63395 (10)	0.0339 (4)	
H8A	0.5834	0.5450	0.6361	0.041*	
H8B	0.7186	0.4646	0.6531	0.041*	
C9	0.4981 (3)	0.41421 (15)	0.68229 (10)	0.0358 (4)	
H9A	0.4775	0.4555	0.7254	0.043*	
H9B	0.3967	0.4052	0.6571	0.043*	
C10	0.5572 (2)	0.31281 (15)	0.70481 (10)	0.0319 (4)	
C11	0.6388 (2)	0.52750 (13)	0.50509 (10)	0.0316 (4)	
H11	0.7222	0.5723	0.5257	0.038*	
C12	0.7038 (3)	0.47736 (15)	0.44038 (11)	0.0368 (4)	
C13	0.4960 (2)	0.59112 (14)	0.48985 (11)	0.0341 (4)	
H13A	0.4181	0.5502	0.4631	0.041*	
H13B	0.4470	0.6121	0.5354	0.041*	
C14	0.6724 (3)	0.31561 (18)	0.76475 (11)	0.0470 (5)	
H14A	0.7063	0.2464	0.7760	0.070*	
H14B	0.6226	0.3460	0.8067	0.070*	
H14C	0.7639	0.3563	0.7509	0.070*	
C15	0.9019 (2)	0.20263 (16)	0.62710 (11)	0.0378 (5)	
H15A	0.9661	0.1412	0.6315	0.057*	
H15B	0.8679	0.2247	0.6745	0.057*	
H15C	0.9642	0.2564	0.6047	0.057*	
C2'	0.4037 (2)	0.74860 (14)	0.43624 (11)	0.0334 (4)	
H2'	0.3648	0.7720	0.4838	0.040*	
C3'	0.4645 (2)	0.84025 (14)	0.39714 (10)	0.0299 (4)	
C4'	0.5552 (2)	0.90996 (15)	0.43477 (11)	0.0352 (4)	
H4'	0.5774	0.8975	0.4836	0.042*	
C5'	0.6133 (2)	0.99567 (15)	0.40354 (11)	0.0359 (4)	
H5'	0.6759	1.0419	0.4301	0.043*	
C6'	0.5793 (2)	1.01419 (15)	0.33243 (11)	0.0336 (4)	
H6'	0.6179	1.0738	0.3100	0.040*	
C7'	0.4899 (2)	0.94620 (15)	0.29450 (10)	0.0329 (4)	
H7'	0.4676	0.9593	0.2458	0.040*	
C8'	0.4317 (2)	0.85882 (14)	0.32608 (10)	0.0306 (4)	
C9'	0.3252 (2)	0.79086 (16)	0.28324 (10)	0.0365 (4)	
H9'1	0.3710	0.7807	0.2353	0.044*	
H9'2	0.2216	0.8242	0.2773	0.044*	
C10'	0.3021 (3)	0.68777 (16)	0.31904 (11)	0.0398 (5)	
H10A	0.2149	0.6508	0.2956	0.048*	
H10B	0.3991	0.6468	0.3142	0.048*	
C11'	0.2645 (2)	0.70309 (14)	0.39664 (10)	0.0346 (4)	
H11A	0.2364	0.6371	0.4184	0.042*	
H11B	0.1725	0.7488	0.4012	0.042*	

### Atomic displacement parameters (Å^2^)

**Table:**

	*U*^11^	*U*^22^	*U*^33^	*U*^12^	*U*^13^	*U*^23^
N1	0.0365 (9)	0.0270 (8)	0.0461 (9)	0.0004 (7)	−0.0010 (7)	0.0058 (7)
O1	0.0324 (6)	0.0278 (6)	0.0343 (6)	0.0068 (6)	0.0029 (5)	−0.0040 (5)
O2	0.0362 (7)	0.0304 (7)	0.0421 (7)	0.0045 (6)	0.0123 (6)	0.0074 (6)
O3	0.0659 (11)	0.0398 (8)	0.0465 (9)	0.0060 (8)	0.0143 (8)	0.0147 (7)
C1	0.0326 (9)	0.0326 (9)	0.0299 (8)	−0.0051 (8)	0.0050 (7)	0.0005 (7)
C2	0.0499 (12)	0.0284 (9)	0.0383 (10)	−0.0087 (9)	0.0034 (9)	0.0038 (8)
C3	0.0433 (11)	0.0207 (8)	0.0424 (11)	0.0012 (8)	−0.0013 (9)	0.0006 (8)
C4	0.0293 (9)	0.0231 (8)	0.0333 (9)	0.0058 (7)	0.0009 (7)	−0.0009 (7)
C5	0.0229 (8)	0.0237 (8)	0.0311 (8)	0.0018 (7)	0.0010 (7)	−0.0026 (7)
C6	0.0226 (8)	0.0251 (8)	0.0347 (9)	−0.0021 (7)	−0.0016 (7)	0.0040 (7)
C7	0.0246 (8)	0.0195 (8)	0.0350 (9)	−0.0003 (7)	−0.0022 (7)	0.0006 (7)
C8	0.0423 (11)	0.0220 (8)	0.0376 (10)	−0.0012 (8)	−0.0042 (8)	−0.0033 (8)
C9	0.0395 (11)	0.0324 (10)	0.0355 (10)	0.0075 (9)	0.0057 (8)	−0.0070 (8)
C10	0.0338 (10)	0.0320 (9)	0.0299 (9)	0.0001 (8)	0.0051 (8)	−0.0013 (7)
C11	0.0308 (9)	0.0213 (8)	0.0427 (10)	−0.0030 (7)	−0.0009 (8)	0.0039 (8)
C12	0.0364 (10)	0.0291 (9)	0.0448 (11)	−0.0018 (8)	0.0051 (8)	0.0089 (8)
C13	0.0346 (10)	0.0239 (8)	0.0438 (10)	0.0001 (8)	−0.0017 (8)	0.0043 (8)
C14	0.0593 (14)	0.0448 (12)	0.0369 (10)	−0.0012 (11)	−0.0121 (10)	−0.0034 (9)
C15	0.0315 (10)	0.0373 (10)	0.0444 (11)	0.0078 (8)	−0.0088 (8)	0.0016 (8)
C2'	0.0320 (10)	0.0286 (9)	0.0395 (10)	0.0020 (8)	−0.0002 (8)	0.0009 (8)
C3'	0.0257 (8)	0.0277 (9)	0.0364 (9)	0.0022 (7)	−0.0014 (8)	0.0005 (7)
C4'	0.0334 (10)	0.0326 (10)	0.0395 (10)	−0.0022 (8)	−0.0060 (8)	0.0041 (8)
C5'	0.0299 (9)	0.0312 (9)	0.0466 (11)	−0.0040 (8)	−0.0008 (8)	0.0013 (8)
C6'	0.0263 (9)	0.0292 (9)	0.0454 (10)	0.0008 (8)	0.0033 (8)	0.0070 (8)
C7'	0.0300 (9)	0.0350 (10)	0.0338 (9)	0.0037 (8)	0.0022 (8)	0.0041 (8)
C8'	0.0257 (9)	0.0290 (9)	0.0372 (10)	0.0047 (7)	0.0033 (8)	−0.0031 (8)
C9'	0.0335 (10)	0.0414 (11)	0.0346 (9)	−0.0054 (9)	0.0026 (8)	−0.0099 (8)
C10'	0.0389 (11)	0.0366 (10)	0.0439 (11)	−0.0079 (9)	0.0013 (9)	−0.0101 (9)
C11'	0.0334 (9)	0.0290 (9)	0.0415 (10)	−0.0010 (8)	0.0005 (8)	0.0021 (8)

### Geometric parameters (Å, °)

**Table:**

N1—C13	1.448 (2)	C11—C12	1.489 (3)
N1—C2'	1.459 (2)	C11—C13	1.503 (3)
N1—H1N	1.01 (3)	C11—H11	1.0000
O1—C5	1.433 (2)	C13—H13A	0.9900
O1—C4	1.450 (2)	C13—H13B	0.9900
O2—C12	1.342 (2)	C14—H14A	0.9800
O2—C6	1.451 (2)	C14—H14B	0.9800
O3—C12	1.198 (2)	C14—H14C	0.9800
C1—C10	1.324 (3)	C15—H15A	0.9800
C1—C2	1.482 (3)	C15—H15B	0.9800
C1—H1	0.9500	C15—H15C	0.9800
C2—C3	1.536 (3)	C2'—C3'	1.505 (3)
C2—H2A	0.9900	C2'—C11'	1.521 (3)
C2—H2B	0.9900	C2'—H2'	1.0000
C3—C4	1.494 (3)	C3'—C8'	1.384 (3)
C3—H3A	0.9900	C3'—C4'	1.392 (3)
C3—H3B	0.9900	C4'—C5'	1.365 (3)
C4—C5	1.457 (2)	C4'—H4'	0.9500
C4—C15	1.485 (3)	C5'—C6'	1.387 (3)
C5—C6	1.481 (2)	C5'—H5'	0.9500
C5—H5	1.0000	C6'—C7'	1.374 (3)
C6—C7	1.523 (2)	C6'—H6'	0.9500
C6—H6	1.0000	C7'—C8'	1.387 (3)
C7—C11	1.522 (2)	C7'—H7'	0.9500
C7—C8	1.523 (3)	C8'—C9'	1.506 (3)
C7—H7	1.0000	C9'—C10'	1.529 (3)
C8—C9	1.523 (3)	C9'—H9'1	0.9900
C8—H8A	0.9900	C9'—H9'2	0.9900
C8—H8B	0.9900	C10'—C11'	1.505 (3)
C9—C10	1.489 (3)	C10'—H10A	0.9900
C9—H9A	0.9900	C10'—H10B	0.9900
C9—H9B	0.9900	C11'—H11A	0.9900
C10—C14	1.492 (3)	C11'—H11B	0.9900
			
C13—N1—C2'	113.94 (16)	C7—C11—H11	108.4
C13—N1—H1N	113.4 (14)	O3—C12—O2	121.07 (19)
C2'—N1—H1N	113.0 (14)	O3—C12—C11	128.96 (19)
C5—O1—C4	60.70 (11)	O2—C12—C11	109.95 (16)
C12—O2—C6	110.53 (15)	N1—C13—C11	111.42 (16)
C10—C1—C2	127.98 (19)	N1—C13—H13A	109.3
C10—C1—H1	116.0	C11—C13—H13A	109.3
C2—C1—H1	116.0	N1—C13—H13B	109.3
C1—C2—C3	109.79 (16)	C11—C13—H13B	109.3
C1—C2—H2A	109.7	H13A—C13—H13B	108.0
C3—C2—H2A	109.7	C10—C14—H14A	109.5
C1—C2—H2B	109.7	C10—C14—H14B	109.5
C3—C2—H2B	109.7	H14A—C14—H14B	109.5
H2A—C2—H2B	108.2	C10—C14—H14C	109.5
C4—C3—C2	111.31 (15)	H14A—C14—H14C	109.5
C4—C3—H3A	109.4	H14B—C14—H14C	109.5
C2—C3—H3A	109.4	C4—C15—H15A	109.5
C4—C3—H3B	109.4	C4—C15—H15B	109.5
C2—C3—H3B	109.4	H15A—C15—H15B	109.5
H3A—C3—H3B	108.0	C4—C15—H15C	109.5
O1—C4—C5	59.08 (11)	H15A—C15—H15C	109.5
O1—C4—C15	113.32 (15)	H15B—C15—H15C	109.5
C5—C4—C15	122.12 (16)	N1—C2'—C3'	108.01 (15)
O1—C4—C3	115.67 (15)	N1—C2'—C11'	115.82 (16)
C5—C4—C3	116.57 (16)	C3'—C2'—C11'	110.19 (16)
C15—C4—C3	116.63 (17)	N1—C2'—H2'	107.5
O1—C5—C4	60.22 (11)	C3'—C2'—H2'	107.5
O1—C5—C6	118.22 (15)	C11'—C2'—H2'	107.5
C4—C5—C6	125.54 (16)	C8'—C3'—C4'	118.90 (17)
O1—C5—H5	114.0	C8'—C3'—C2'	122.89 (17)
C4—C5—H5	114.0	C4'—C3'—C2'	118.20 (17)
C6—C5—H5	114.0	C5'—C4'—C3'	121.92 (19)
O2—C6—C5	105.81 (15)	C5'—C4'—H4'	119.0
O2—C6—C7	105.04 (14)	C3'—C4'—H4'	119.0
C5—C6—C7	114.46 (14)	C4'—C5'—C6'	118.93 (19)
O2—C6—H6	110.4	C4'—C5'—H5'	120.5
C5—C6—H6	110.4	C6'—C5'—H5'	120.5
C7—C6—H6	110.4	C7'—C6'—C5'	119.91 (18)
C11—C7—C6	101.64 (14)	C7'—C6'—H6'	120.0
C11—C7—C8	112.93 (15)	C5'—C6'—H6'	120.0
C6—C7—C8	117.69 (15)	C6'—C7'—C8'	121.19 (18)
C11—C7—H7	108.0	C6'—C7'—H7'	119.4
C6—C7—H7	108.0	C8'—C7'—H7'	119.4
C8—C7—H7	108.0	C3'—C8'—C7'	119.15 (18)
C7—C8—C9	115.68 (16)	C3'—C8'—C9'	122.00 (17)
C7—C8—H8A	108.4	C7'—C8'—C9'	118.71 (17)
C9—C8—H8A	108.4	C8'—C9'—C10'	111.81 (17)
C7—C8—H8B	108.4	C8'—C9'—H9'1	109.3
C9—C8—H8B	108.4	C10'—C9'—H9'1	109.3
H8A—C8—H8B	107.4	C8'—C9'—H9'2	109.3
C10—C9—C8	114.03 (16)	C10'—C9'—H9'2	109.3
C10—C9—H9A	108.7	H9'1—C9'—H9'2	107.9
C8—C9—H9A	108.7	C11'—C10'—C9'	109.47 (16)
C10—C9—H9B	108.7	C11'—C10'—H10A	109.8
C8—C9—H9B	108.7	C9'—C10'—H10A	109.8
H9A—C9—H9B	107.6	C11'—C10'—H10B	109.8
C1—C10—C9	119.88 (18)	C9'—C10'—H10B	109.8
C1—C10—C14	125.72 (19)	H10A—C10'—H10B	108.2
C9—C10—C14	114.40 (17)	C10'—C11'—C2'	111.16 (17)
C12—C11—C13	113.08 (17)	C10'—C11'—H11A	109.4
C12—C11—C7	103.38 (14)	C2'—C11'—H11A	109.4
C13—C11—C7	114.96 (16)	C10'—C11'—H11B	109.4
C12—C11—H11	108.4	C2'—C11'—H11B	109.4
C13—C11—H11	108.4	H11A—C11'—H11B	108.0
			
C10—C1—C2—C3	−109.7 (2)	C8—C7—C11—C13	−80.1 (2)
C1—C2—C3—C4	45.8 (2)	C6—O2—C12—O3	−176.92 (19)
C5—O1—C4—C15	−114.62 (18)	C6—O2—C12—C11	2.1 (2)
C5—O1—C4—C3	106.90 (17)	C13—C11—C12—O3	33.4 (3)
C2—C3—C4—O1	−155.15 (16)	C7—C11—C12—O3	158.4 (2)
C2—C3—C4—C5	−88.5 (2)	C13—C11—C12—O2	−145.45 (17)
C2—C3—C4—C15	67.8 (2)	C7—C11—C12—O2	−20.5 (2)
C4—O1—C5—C6	116.94 (18)	C2'—N1—C13—C11	−176.27 (16)
C15—C4—C5—O1	99.73 (18)	C12—C11—C13—N1	−69.3 (2)
C3—C4—C5—O1	−105.36 (17)	C7—C11—C13—N1	172.34 (15)
O1—C4—C5—C6	−105.11 (19)	C13—N1—C2'—C3'	176.64 (16)
C15—C4—C5—C6	−5.4 (3)	C13—N1—C2'—C11'	−59.3 (2)
C3—C4—C5—C6	149.53 (17)	N1—C2'—C3'—C8'	108.4 (2)
C12—O2—C6—C5	138.80 (16)	C11'—C2'—C3'—C8'	−19.0 (2)
C12—O2—C6—C7	17.36 (19)	N1—C2'—C3'—C4'	−72.8 (2)
O1—C5—C6—O2	56.9 (2)	C11'—C2'—C3'—C4'	159.81 (17)
C4—C5—C6—O2	128.86 (18)	C8'—C3'—C4'—C5'	−0.3 (3)
O1—C5—C6—C7	172.03 (14)	C2'—C3'—C4'—C5'	−179.21 (18)
C4—C5—C6—C7	−115.99 (19)	C3'—C4'—C5'—C6'	0.7 (3)
O2—C6—C7—C11	−28.52 (17)	C4'—C5'—C6'—C7'	−0.6 (3)
C5—C6—C7—C11	−144.12 (16)	C5'—C6'—C7'—C8'	0.3 (3)
O2—C6—C7—C8	−152.39 (15)	C4'—C3'—C8'—C7'	−0.1 (3)
C5—C6—C7—C8	92.0 (2)	C2'—C3'—C8'—C7'	178.76 (17)
C11—C7—C8—C9	145.45 (17)	C4'—C3'—C8'—C9'	−175.66 (18)
C6—C7—C8—C9	−96.5 (2)	C2'—C3'—C8'—C9'	3.2 (3)
C7—C8—C9—C10	78.9 (2)	C6'—C7'—C8'—C3'	0.1 (3)
C2—C1—C10—C9	168.58 (19)	C6'—C7'—C8'—C9'	175.83 (18)
C2—C1—C10—C14	−10.4 (3)	C3'—C8'—C9'—C10'	−17.3 (3)
C8—C9—C10—C1	−99.0 (2)	C7'—C8'—C9'—C10'	167.10 (17)
C8—C9—C10—C14	80.1 (2)	C8'—C9'—C10'—C11'	47.4 (2)
C6—C7—C11—C12	29.12 (18)	C9'—C10'—C11'—C2'	−66.1 (2)
C8—C7—C11—C12	156.16 (16)	N1—C2'—C11'—C10'	−72.9 (2)
C6—C7—C11—C13	152.84 (16)	C3'—C2'—C11'—C10'	50.1 (2)

### Hydrogen-bond geometry (Å, °)

**Table:**

*D*—H···*A*	*D*—H	H···*A*	*D*···*A*	*D*—H···*A*
N1—H1N···O3	1.01 (3)	2.32 (3)	2.992 (2)	123.5 (18)
